# Rosiglitazone and Myocardial Infarction in Patients Previously Prescribed Metformin

**DOI:** 10.1371/journal.pone.0006080

**Published:** 2009-06-27

**Authors:** Colin R. Dormuth, Malcolm Maclure, Greg Carney, Sebastian Schneeweiss, Ken Bassett, James M. Wright

**Affiliations:** 1 Therapeutics Initiative, University of British Columbia, Vancouver, Canada; 2 Department of Anesthesiology, Pharmacology and Therapeutics, The University of British Columbia. Vancouver, Canada; 3 School of Health Information Science, University of Victoria, Victoria, Canada; 4 Pharmaceutical Services Division, British Columbia Ministry of Health, Victoria, Canada; 5 Division of Pharmacoepidemiology and Pharmacoeconomics, Brigham and Women's Hospital, Harvard Medical School, Boston, Massachusetts, United States of America; 6 Department of Epidemiology, Harvard School of Public Health, Boston, Massachusetts, United States of America; 7 Department of Medicine, University of British Columbia, Vancouver, Canada; Canadian Agency for Drugs and Technologies in Health, Canada

## Abstract

**Objective:**

Rosiglitazone was found associated with approximately a 43% increase in risk of acute myocardial infarction (AMI) in a two meta-analyses of clinical trials. Our objective is to estimate the magnitude of the association in real-world patients previously treated with metformin.

**Research Design and Methods:**

We conducted a nested case control study in British Columbia using health care databases on 4.3 million people. Our cohort consisted of 158,578 patients with Type 2 diabetes who used metformin as first-line drug treatment. We matched 2,244 cases of myocardial infarction (AMI) with up to 4 controls. Conditional logistic regression models were used to estimate matched odds ratios for AMI associated with treatment with rosiglitazone, pioglitazone and sulfonylureas.

**Results:**

In our cohort of prior metformin users, adding rosiglitazone for up to 6 months was not associated with an increased risk of AMI compared to adding a sulfonylurea (odds ratio [OR] 1.38; 95% confidence interval [CI], 0.91–2.10), or compared to adding pioglitazone (OR for rosi versus pio 1.41; 95% CI, 0.74–2.66). There were also no significant differences between rosiglitazone, pioglitazone and sulfonylureas for longer durations of treatment. Though not significantly different from sulfonylureas, there was a transient increase in AMI risk associated with the first 6 months of treatment with a glitazone compared to not using the treatment (OR 1.53; 95% CI, 1.13–2.07)

**Conclusions:**

In our British Columbia cohort of patients who received metformin as first-line pharmacotherapy for Type 2 diabetes mellitus, further treatment with rosiglitazone did not increase the risk of AMI compared to patients who were treated with pioglitazone or a sulfonylurea. Though not statistically significantly different compared from each other, an increased risk of AMI observed after starting rosiglitazone or sulfonylureas is a matter of concern that requires more research.

## Introduction

Rosiglitazone (Avandia®) is a peroxisome-proliferator-activated receptor γ agonist used to treat patients with Type 2 diabetes mellitus. The medication is taken to lower and control blood glucose. In June 2007, meta-analysis of randomized clinical trial data found a statistically significant 42%–43% increase in risk of myocardial infarction (AMI) associated with rosiglitazone treatment [1], [2]. The meta-analyses did not show an increased risk of AMI compared separately to placebo, metformin, sulfonylureas or insulin. Epidemiologic studies have reported conflicting results. One study reported an increased risk of AMI with rosiglitazone treatment compared to treatment with metformin and sulfonylurea monotherapy in older patients with diabetes [3]. Another study did not find a significant association with AMI compared to treatment with metformin or sulfonylurea [4]. The relationship between rosiglitazone and AMI remains controversial. At least one study showed that the Nissen meta-analysis could have overestimated the cardiovascular risks of rosiglitazone by excluding trials with zero events [5].

We investigated the association between AMI and treatment with rosiglitazone, pioglitazone and sulfonylureas in patients who added or switched to these drugs from metformin as first-line drug treatment. British Columbia (BC), Canada, provided a unique opportunity for this kind of analysis because, since late 1995, its comprehensive PharmaNet database has captured all prescriptions dispensed at community pharmacies to its large and stable population (4.3 million in 2006). Since that time, approximately 77% of patients with Type 2 diabetes mellitus were started on metformin as first-line drug treatment.

## Methods

We obtained ethics approval from the University of British Columbia Clinical Research Ethics Board and we were not required to obtain informed consent from patients included in the study. All data were analyzed anonymously.

### Data

The PharmaNet database contains all prescriptions, regardless of payer, dispensed at community pharmacies in BC since the autumn of 1995. We expect that underreporting and misclassification are very low because the PharmaNet system performs data quality checks. Prescriptions were linked by unique personal health number to BC Ministry of Health databases for hospitalizations, medical services registration, and family income. Data on hospitalizations were collected by the Canadian Institute for Health Information, which collects hospitals data for all Canadian provinces, including Ontario where the data have been evaluated for accuracy [6]. The completeness of similar databases in other North American jurisdictions has been studied [6]–[10] but we are unaware of any such analyses in British Columbia.

### Source Population and Cohort

We conducted a nested case-control study. The source population included all residents of BC at any time between January 1997 and March 2007 who were registered for provincial medical coverage for at least one year. Federally insured patients (aboriginals, prisoners and military personnel), 4% of the provincial population, were excluded from the source population because we did not have permission to use those data. The source population numbered 4.1 million in 2006 [11]. We assembled a cohort of patients from the source population who initiated metformin between January 1, 1997 and March 31, 2007. Initiation was defined as a pharmacy dispensing for metformin without another metformin dispensing in the previous 365 days. Patients were excluded if they received other oral antidiabetic medications or insulin within 365 days before starting metformin, or if they emigrated from BC or died prior to May 1, 2003.

The study period ran from May 1, 2003 to March 31, 2007. The study period was chosen based on the availability of family-specific income data starting in May of 2003. Glitazones cost more than metformin and sulfonylureas, and the BC Ministry of Health covered them only after failure or intolerance to metformin and two sulfonylureas. We adjusted for income because use of glitazones without insurance coverage could be correlated with a patient's socioeconomic status and risk of AMI.

### Myocardial Infarction Cases

We extracted patients from the BC Ministry of Health hospitalizations database who were admitted to hospital with acute myocardial infarction (ICD-9 410) recorded as the primary reason for the admission. A validation study of the U.S. Medicare database found that hospitalizations with an ICD-9 code of 410 as the primary or secondary diagnosis code had a positive predictive value for AMI of 0.94 [12]. Cohort members were eligible for selection as cases after the latest date of May 1, 2003 or initiation of metformin, and until the earliest date of the occurrence of the outcome, death, emigration from BC, or March 31, 2007. We estimated that 2,100 AMI cases and 8,400 controls would be needed to observe an odds ratio of 1.40 with 80% power, a Type-I error of 0.05, and an exposure prevalence of 3.5%.

### Controls and Matching

Patients were eligible to be controls if they were in the cohort of metformin initiators and were still contributing person-time at risk for the outcome at the time of the AMI of their matching case. Each case was demographically matched with up to 4 controls. Patients were matched on age (in 5-year categories), sex, number of family members, enrollment in supplemental health coverage, and family income in bands of $5,000 for incomes under $100,000, and bands of $25,000 for incomes above $100,000. Number of family members and income were used to match cases to controls that had similar abilities to pay for prescription drugs. Controls were not matched on cardiovascular risk factors as these could be intermediates in the causal pathway between medication use and AMI. Controls were selected randomly using incidence density sampling from patients with the same matching factors as the cases in ascending order of random number assignment.

### Exposure to Oral Antidiabetic Medications

We evaluated exposure to the glitazones (rosiglitazone and pioglitazone) and the sulfonylureas (acetohexamide, chlorpropamide, gliclazide, glimepiride, glyburide, tolbutamide). We extracted all prescriptions for these drugs before the event date (cases) or index date (controls). We divided exposure into current accumulated use and past use. Exposure within 90 days of the index date was defined as current exposure. All preceding exposure was summed as current cumulative exposure so long as no interruption in use of greater than 90 days occurred. Exposure prior to any 90 day interruption was categorized as past exposure.

### Case-Control Analysis

We used conditional logistic regression models to estimate the matched odds ratios for AMI. We estimated odds ratios related to 5 exposures: rosiglitazone, pioglitazone, either rosiglitazone or pioglitazone, any sulfonylurea, and glyburide, which was the most commonly prescribed sulfonylurea in BC. Associations with exposure duration were modeled using predefined categories of 1 to 6, 7 to 12, 13 to 24, and >24 months of current cumulative exposure. Past exposure was modeled as a binary indicator variable.

In addition to the demographic matching factors, odds ratios were adjusted for duration of diabetes (counted from the earliest date of a diagnosis for diabetes or initiation of metformin) and the following covariates within 5 years of the index date: congestive heart failure (CHF: hospitalization for ICD-9 428 or a physician visit for same plus a prescription for furosemide), angiography, coronary artery bypass graft (CABG), percutaneous transluminal angioplasty (PTCA), ischemic stroke (hospitalization for ICD-9 433, 434 or 436), transient ischemic attack (hospitalization for ICD-9 435), angina (ICD-9 412–414), prior AMI, renal disease (ICD-9 584–586, 403–404). The following covariates were measured and adjusted for within one year of the index date: Romano comorbidity score (meant to adjust for confounding by concomitant illnesses by assigning weights to a patient's ICD-9 diagnoses and summing those weights into a single score) [13], exposure to nitrates, statins, angiotensen II converting enzyme inhibitors or receptor blockers, thiazide diuretics, calcium channel blockers, beta blockers, clopidogrel, digoxin, warfarin, insulin, and past use of metformin, glitazones and sulfonylureas, and total number of distinct drugs taken. Including previous drug use enabled the analysis to adjust for past treatment failures and successes.

## Results

There were 189,563 patients from the source population who initiated metformin between January 1, 1997 and March 31, 2007. Of those, 158,578 remained eligible for cohort entry. We identified 2,244 cases of acute myocardial infarction in the cohort during the follow-up period. The cases were matched to 8,903 controls. Characteristics of the cases and controls are shown in Table 1. As expected, the groups were demographically similar and differed significantly with respect to cardiovascular risk factors. A higher proportion of cases had renal disease, prior AMI, angina, CHF, and prior procedures such as CABG, coronary catheterization and PTCA. There were 7.7% of cases and 7.1% of controls that used a glitazone within a year of their index date. Similar proportions of cases and controls used metformin in the previous year (80%). Forty percent of cases and 32% of controls received a sulfonylurea.

**Table 1:** Characteristics of Type II Diabetes Patients with Myocardial Infarction and Their Matched Controls*.

Variable	Cases of Acute		
	Myocardial Infarction	Controls	Odds Ratio
	(N = 2,244)	(N = 8,903)	(95% CI)
Age,mean(SD),	70 (12)	70 (12)	1.00 (0.99–1.01)
Female (%)	806 (36)	3,201 (36)	1.00 (0.90–1.11)
Income category (%)†			
$0–$24,999	605 (27)	2,369 (27)	1.02 (0.92–1.14)
$25,000–$49,999	759 (34)	2,978 (33)	1.02 (0.93–1.12)
$50,000–$74,999	338 (15)	1,363 (15)	0.98 (0.87–1.11)
$75,000–$99,999	121 (5)	482 (5)	0.99 (0.82–1.20)
> = $100,000	72 (3)	271 (3)	1.04 (0.81–1.33)
Unknown	349 (16)	1,440 (16)	0.96 (0.85–1.08)
Romano score mean (SD)‡	2.0 (1.7)	1.5 (1.3)	1.18 (1.15–1.21)
Diabetes duration, mean (SD), y	6.9 (4.4)	6.4 (4.2)	1.03 (1.02–1.04)
Renal disease	142 (6)	244 (3)	1.97 (1.64–2.37)
Acute myocardial infarction§	190 (8)	293 (3)	2.19 (1.87–2.57)
Angina§	1,201 (54)	2,854 (32)	2.18 (1.99–2.39)
Congestive heart failure§	788 (35)	1,964 (22)	1.85 (1.68–2.05)
Coronary artery bypass graft§	43 (2)	209 (2)	0.88 (0.64–1.20)
Coronary catheterization§	363 (16)	742 (8)	1.81 (1.60–2.05)
PTCA§	173 (8)	288 (3)	2.01 (1.69–2.39)
Drug use in previous year∥			
Glitazone	173 (8)	631 (7)	1.10 (0.92–1.30)
Metformin	1,798 (80)	7,081 (80)	1.02 (0.91–1.14)
Sulfonylurea	907 (40)	2,825 (32)	1.38 (1.26–1.51)
ACE Inhibitor	1,241 (55)	4,565 (51)	1.12 (1.02–1.22)
NSAIDs	606 (27)	2,170 (24)	1.10 (1.00–1.22)
Beta blockers	1,641 (73)	5,985 (67)	1.30 (1.17–1.44)
Thiazide diuretics	743 (33)	2,986 (34)	1.00 (0.91–1.10)
Digoxin	238 (11)	515 (6)	1.67 (1.44–1.94)
Spironolactone	165 (7)	365 (4)	1.64 (1.38–1.94)
Statins	1,104 (49)	4,096 (46)	1.11 (1.02–1.22)
Calcium channel blockers	735 (33)	2,157 (24)	1.42 (1.29–1.57)
Clopidogrel	248 (11)	392 (4)	2.09 (1.81–2.42)
No. drugs prescribed (SD)	11 (6.4)	9 (5.4)	1.05 (1.04–1.06)
No. Physician Visits (SD)	21 (17)	18 (14.6)	1.01 (1.00–1.01)

* Odds ratios have been adjusted for matching variables (age in 5-year groupings, sex, family income band in $5,000 dollar increments, number of family members, and existence of supplemental coverage). CI denotes confidence interval.

† Net family income band in Canadian dollars from the most recent federal income tax return (1 Canadian dollar = 1.2 US dollars).

‡ Romano commorbidity score calculated using data from 365 days prior to the index date.

§ History within 5 years prior to the index date.

∥ Dispensing of a drug within 365 days prior to index date.

Baseline characteristics in the study population were also compared to identify potential confounders associated with treatment. Age, sex and income were significantly associated with exposure to glitazones (Table 2), confirming the value of matching on those demographic factors. Patients exposed to glitazones and sulfonylureas had diabetes for approximately one year longer on average than unexposed patients. Prior AMI was associated with glitazone exposure but potential for confounding was low because the prevalence was low (3% of exposed patients and 4% of unexposed patients).

**Table 2:** Characteristics of Study Patients (Cases and Controls).

Variable	Rosiglitazone		Pioglitazone		Sulfonylurea	
	Exposed	Unexposed	Exposed	Unexposed	Exposed	Unexposed
	(N = 462)	(N = 10,685)	(N = 235)	(N = 10,912)	(N = 1,612)	(N = 9,535)
Age, mean (SD)	66 (11)	70 (12)	66 (12)	70 (12)	69 (12)	70 (12)
Female (%)	113 (24)	3,894 (36)	69 (29)	3,938 (36)	581 (36)	3,426 (36)
Income category (%)*						
$0–$24,999	85 (18)	2,889 (27)	40 (17)	2,934 (27)	477 (30)	2,497 (26)
$25,000–$74,999	147 (32)	3,590 (34)	82 (35)	3,655 (33)	521 (32)	3,216 (34)
$75,000–$99,999	116 (25)	1,585 (15)	42 (18)	1,659 (15)	238 (15)	1,463 (15)
> = $100,000	52 (11)	551 (5)	31 (13)	572 (5)	82 (5)	521 (5)
Unknown	37 (8)	318 (3)	19 (8)	324 (3)	48 (3)	295 (3)
Romano score mean (SD)†	1.7 (1.4)	1.6 (1.4)	1.5 (1.1)	1.6 (1.4)	1.8 (1.5)	1.6 (1.4)
Diabetes duration, mean (SD), y	7.7 (4)	7.5 (4.2)	7.7 (4)	6.5 (4.2)	7.5 (4.2)	6.3 (4.2)
Renal disease	11 (2)	375 (4)	4 (2)	382 (4)	88 (5)	298 (3)
Acute myocardial infarction‡	7 (2)	476 (4)	11 (5)	472 (4)	67 (4)	416 (4)
Angina‡	175 (38)	3,880 (36)	90 (38)	3,965(36)	595 (37)	3,460 (36)
Congestive heart failure‡	108 (23)	2,644 (25)	55 (23)	2,697 (25)	438 (27)	2,314 (24)
Coronary artery bypass graft‡	9 (2)	243 (2)	8 (3)	244 (2)	32 (2)	220 (2)
Coronary catheterization‡	40 (9)	1,065 (10)	28 (12)	1,077 (10)	163 (10)	942 (10)
PTCA‡	16 (3)	445 (4)	10 (4)	451 (4)	76 (5)	385 (4)
Drug Use in Past Year§						
Glitazone	460 (100)	344 (3)	235 (100)	571 (5)	206 (13)	598 (6)
Metformin	361 (78)	8,518 (80)	188 (80)	8,691 (80)	1,309 (81)	7,570(79)
Sulfonylurea	267 (58)	3,465 (32)	128 (54)	3,604 (33)	1,612 (100)	2,121 (22)
ACE Inhibitor	262 (57)	5,544 (52)	145 (62)	5,661 (52)	962 (57)	4,880 (51)
NSAIDs	111 (24)	2,665 (25)	51 (22)	2,725 (25)	416 (26)	2,360 (25)
Beta blockers	352 (76)	7,274 (68)	191 (81)	7,435 (68)	1,214 (75)	6,412 (67)
Thiazide diuretics	207 (45)	3,522 (33)	80 (34)	3,649 (33)	593 (37)	3,136 (33)
Digoxin	24 (5)	729 (7)	17 (7)	736 (7)	123 (8)	630 (7)
Spironolactone	27 (6)	503 (5)	14 (6)	516 (5)	85 (5)	445 (5)
Statins	289 (63)	4,911(46)	146 (62)	5,054 (46)	854 (53)	4,346 (46)
Calcium channel blockers	140 (30)	2,752 (26)	53 (23)	2,839 (26)	455 (28)	2,437 (26)
Clopidogrel	25 (5)	615 (6)	13 (6)	627 (6)	115 (7)	525 (6)
No. drugs prescribed (SD)	11 (5.2)	9 (5.7)	11 (4.4)	9 (5.7)	11 (5.3)	9 (5.7)
No. Physician Visits (SD)	19 (15.2)	18 (15.2)	19 (13.9)	18 (15.2)	19 (14.9)	18 (15.2)

* Net family income in Canadian dollars (1 Canadian dollar = 1.2 US dollars).

† Romano commorbidity score calculated using data from 365 days prior to the index date.

‡ History within 5 years prior to the index date.

§ Dispensing of a drug within 365 days prior to index date.

As shown in Table 3, the risk of AMI in the first 6 months of treatment with rosiglitazone was not significantly different compared to 6 months of treatment with sulfonylureas (odds ratio 1.38; 95% CI, 0.91–2.10) or pioglitazone (odds ratio 1.41; 95% CI, 0.74–2.66). There were also no significant differences between OADs for longer durations of use. However, within-drug analyses which studied the association between adding a treatment compared to not adding it showed transient increases in AMI risk (Table 4). Addition of glitazone therapy for up to 6 months duration was associated with a 50% increased risk of AMI compared to not adding a glitazone (odds ratio 1.53; 95% confidence interval [CI] 1.13–2.07). This association was observed with rosiglitazone (odds ratio 1.71; 95% CI, 1.19–2.46) but not for pioglitazone (odds ratio 1.21; 95% CI, 0.72–2.04) although the two confidence intervals overlapped. Exposures longer than 6 months were not associated with significant changes in risk of AMI for either rosiglitazone or pioglitazone, separately or combined. Unlike the glitazones, however, elevated risk associated with sulfonylurea use was also observed for treatment durations between 7 months and a year (odds ratio 1.24; 95% CI, 1.01–1.52) and over 24 months (odds ratio 1.45; 95% CI, 1.17–1.81). A separate analysis of glyburide alone showed similar results as for all sulfonylureas as a class.

**Table 3:** Risk of Myocardial Infarction for Rosiglitazone Compared to Pioglitazone and Sulfonylureas in Patients Who Received Metformin as First-Line Drug Treatment.

Rosiglitazone Comparator	Current Cumulative Exposure (months)†	Unadjusted Odds Ratio for Rosiglitazone (95% CI)‡ *	Adjusted Odds Ratio for Rosiglitazone (95% CI)§ *
Sulfonylureas	1–6	1.17 (0.79–1.74)	1.38 (0.91–2.10)
	7–12	0.84 (0.50–1.41)	0.75 (0.44–1.27)
	13–24	0.59 (0.33–1.06)	0.76 (0.41–1.38)
	>24	0.60 (0.37–0.96)	0.68 (0.41–1.12)
	Overall	0.81 (0.63–1.03)	0.90 (0.69–1.17)
Pioglitazone	1–6	1.39 (0.76–2.52)	1.41 (0.74–2.66)
	7–12	0.89 (0.40–1.97)	0.95 (0.41–2.22)
	13–24	0.64 (0.28–1.45)	0.68 (0.29–1.60)
	>24	0.88 (0.41–1.89)	0.93 (0.41–2.11)
	Overall	0.97 (0.67–1.40)	1.00 (0.67–1.49)

* CI denotes confidence interval.

† The current cumulative exposure period is the number of months of continuous exposure prior to the event (for cases) or matched index date (controls). Cumulative current exposure includes continuous drug use up until the index date. Exposure that was accumulated prior to any lapse in therapy of greater than 90 days was defined as past exposure.

‡ Odds ratios have been adjusted for matching variables (age in 5-year groupings, sex, family income band in $5,000 dollar increments, number of family members, and existence of supplemental coverage).

§ Odds ratios have been adjusted for (in addition to the matching variables) the time since initiation of metformin, the following within 5 years of the index date: congestive heart failure (hospitalization for ICD-9 428 or a physician visit for same plus a prescription for furosemide), angiography, coronary artery bypass graft, percutaneous transluminal angioplasty, ischemic stroke (hospitalization for ICD-9 433, 434, or 436), transient ischemic attack (hospitalization for ICD-9 435), angina (ICD-9 412–414), prior AMI, renal disease (ICD-9 584–586, 403–404); and the following within one year of index: Romano comorbidity score, exposure to nitrates, statins, angiotensen II converting enzyme inhibitors or receptor blockers, thiazide diuretics, calcium channel blockers, beta blockers, clopidogrel, digoxin, warfarin, insulin, and past use of metformin, glitazones and sulfonylureas.

**Table 4:** Within-Drug Comparison of Glitazone Exposure and Sulfonylurea Exposure in Myocardial Infarction Cases and Matched Controls who received Metformin as First-Line Drug Therapy.

Oral Diabetes Medication	Current Cumulative Exposure (months)†	No. of Cases (n = 2,244)	No. of Controls (n = 8,903)	Unadjusted Odds Ratio‡	Adjusted Odds Ratio (95% CI)§ *
Glitazones	1–6	57	157	1.45	1.53 (1.13–2.07)
	7–12	31	110	1.12	1.00 (0.67–1.47)
	13–24	26	120	0.86	0.97 (0.64–1.48)
	>24	35	160	0.87	0.99 (0.68–1.45)
	Overall	149	547	1.09	1.14 (0.94–1.38)
	Past Use	124	427	1.11	0.93 (0.75–1.14)
Rosiglitazone	1–6	18	64	1.12	1.71(1.19–2.46)
	7–12	10	33	1.20	0.97(0.61–1.54)
	13–24	12	42	1.13	0.83(0.47–1.45)
	>24	11	45	0.97	0.99(0.63–1.55)
	Overall	51	184	1.10	1.14(0.90–1.43)
	Past Use	41	134	1.19	0.94(0.74–1.19)
Pioglitazone	1–6	18	64	1.12	1.21 (0.72–2.04)
	7–12	10	33	1.20	1.02 (0.50–2.07)
	13–24	12	42	1.13	1.21 (0.64–2.30)
	>24	11	45	0.97	1.21 (0.54–2.10)
	Overall	51	184	1.10	1.21 (0.82–1.57)
	Past Use	41	134	1.19	1.21 (0.64–1.29)
Sulfonylureas	1–6	121	368	1.32	1.24 (1.01–1.52)
	7–12	80	257	1.24	1.29 (1.01–1.65)
	13–24	92	303	1.21	1.09 (0.87–1.38)
	>24	107	284	1.52	1.45 (1.17–1.81)
	Overall	400	1,212	1.31	1.26 (1.12–1.43)
	Past Use	585	1,959	1.21	1.09 (0.97–1.21)
Glyburide	1–6	84	255	1.32	1.37 (1.08–1.74)
	7–12	58	181	1.28	1.38 (1.03–1.83)
	13–24	65	212	1.22	1.07 (0.81–1.41)
	>24	73	196	1.49	1.43 (1.11–1.85)
	Overall	280	844	1.32	1.31 (1.13–1.51)
	Past Use	507	1,680	1.21	1.07 (0.96–1.21)

* CI denotes confidence interval.

† The current cumulative exposure period is the number of months of continuous exposure prior to the event (for cases) or matched index date (controls). Cumulative current exposure includes continuous drug use up until the index date. Exposure that was accumulated prior to any lapse in therapy of greater than 90 days was defined as past exposure.

‡ Odds ratios have been adjusted for matching variables (age in 5-year groupins, sex, family income band in $5,000 dollar increments, number of family members, and existence of supplemental coverage).

§ Odds ratios have been adjusted for (in addition to the matching variables) time since initiation of metformin, the following within 5 years of the index date: congestive heart failure, angiography, coronary artery bypass graft, percutaneous transluminal angioplasty, ischemic stroke, transient ischemic attack, angina, prior AMI, renal disease; and the following within one year of index: Romano comorbidity score, exposure to nitrates, statins, angiotensen II converting enzyme inhibitors or receptor blockers, thiazide diuretics, calcium channel blockers, beta blockers, clopidogrel, digoxin, warfarin, insulin, and past use of metformin, glitazones and sulfonylureas.

## Discussion

This study provides comparative data on the relationship between myocardial infarction and treatment with glitazones and sulfonylureas in patients who switched to or added those drugs to first-line treatment with metformin. Adding rosiglitazone treatment did not significantly increase risk of AMI compared to adding pioglitazone or a sulfonylurea. Patients in our study were drawn from the broadest population of glitazone patients studied to date. Our results are generalizable to patients with Type 2 diabetes who received metformin as first-line drug treatment. From our data, and for the 10-year period ending in March 2007, we estimated that metformin was used as first-line drug treatment in 77% of patients with Type 2 diabetes.

There is evidence from trials like ACCORD [14] and UGDP [15] that more intensive hypoglycemic therapy increases cardiovascular risk. The purpose of the analysis in Table 4 was to estimate temporal associations within each drug to see if increased risk was generally associated with more treatment with OADs, an effect that could have been masked in the analysis of drug-to-drug comparisons in Table 3. In Table 4, an increased risk of AMI was not observed with pioglitazone, which is consistent with the null result for pioglitazone and MI reported in a meta-analysis of randomized trials of pioglitazone [16]. However, our power to detect an association was lower because the use of pioglitazone in the source population was half that of rosiglitazone. Also in Table 4, addition of sulfonylurea therapy was associated with a significant 25% increase in risk that appeared to be independent of duration of use. The increase associated with the addition of rosiglitazone or a sulfonylurea could be clinically significant and suggests that either worsening glycemic control (which leads to treatment intensification) increases cardiovascular risk, or, alternatively, that increased risk is a result of more intensive therapy as shown for CV death but not AMI in the ACCORD trial [14]. It is tempting to assume that former explanation is correct, but defending that assumption requires selective use of evidence or at least a clear refutation of evidence from clinical trials, meta-analyses and observational studies that lend merit to the latter explanation. In our opinion, increased risk of cardiovascular events as a consequence of treatment with any OAD is a credible hypothesis that requires more research.

The Nissen meta-analysis reported an overall 43% increase (95% CI, 3%–98%) in AMI events in patients treated with rosiglitazone compared to controls on various treatments including placebo [1]. A direct comparison of our analysis with that overall result is not simple because only 35% of patients in the meta-analysis received metformin compared to 80% of patients in our study in the previous year. However, a subgroup comparison in the Nissen meta-analysis of trials that used metformin as a control showed an odds ratio of 1.14 (95% CI, 0.70–1.86). The odds ratio we observed (1.14 from Table 4) was within the 95% confidence interval of the Nissen meta-analysis and was also not statistically significant. An interim analysis of the Rosiglitazone Evaluated for Cardiovascular Outcome (RECORD) trial also reported a similar hazard ratio to ours for AMI of 1.16 (95% CI, 0.75–1.81) for rosiglitazone (plus metformin or plus sulfonylurea) compared to treatment with metformin plus sulfonylurea [17].

Our results for rosiglitazone are close to the estimates reported in an observational analysis by McAfee. [4]. Their study reported hazard ratios for MI outcomes of 1.19 for rosiglitazone compared to metformin, and a hazard ratio of 0.79 for rosiglitazone compared to sulfonylurea over three years of follow-up. We estimated odds ratios of 1.14 and 0.90, respectively.

In another recent Canadian study of cardiovascular outcomes among patients older than 65 years in Ontario, current treatment with a glitazone (rosiglitazone or pioglitazone) was associated with an odds ratio of 1.40 (95% CI, 1.05–1.86) for AMI compared to patients receiving other OAD medications [3]. The effect size may have been greater than in other studies because, as the authors stated, “our TZD treated population may represent an older and more select population of patients with more advanced diabetes because under Ontario Drug Benefit reimbursement criteria, most of these patients will have failed or had a contraindication to other drugs.”

As with most population-based outcomes studies of prescription drugs, our study was susceptible to channeling bias, which is a type of confounding by indication where marketing leads to sicker patients being more likely to be early users of new drugs. The expected direction of such a bias is to increase the association between glitazones and AMI. We cannot say if our study was more influenced by such forces than other epidemiologic studies of the glitazones, but the 6-month odds ratios for the glitazones (Table 4) increased after multivariable adjustment, which suggests that we likely underestimated the true effect. However, it is still possible that patients with deteriorating health led their physicians to add OADs, and our results could have been biased upwards if such deteriorations were not captured in our claims data but affected AMI risk. Specifically, among patients with diabetes who fluctuate between periods of good and poor control of their blood sugar, glitazones would be initiated in poor periods when risk of AMI might be transiently higher. Therefore, the transient elevation in AMI risk after initiation of rosiglitazone may, at least in part, be due to confounding by indication. Direct comparison of glitazone starters and sulfonylurea starters would be less biased because both classes of drugs would tend to be initiated in periods of poor control. We expect that our within-drug analysis of adding/switching treatment versus not adding/switching treatment (Table 4) would be more vulnerable to this kind of bias than our analysis using comparator drugs (Table 3).

Exposure definitions (current or past use) could not be measured with certainty since dispensing records were used rather than a direct measure of consumption. Some patients dispensed medications may not have taken their drug, causing them to mistakenly be classified as exposed instead of unexposed. So long as the sensitivity and specificity of the exposure definitions were the same in cases and controls, any plausible error rate in classifying exposed patients as unexposed would have biased our estimates towards the null.

Although Nissen's meta-analysis is persuasive, the generalizability of clinical trial results to the general population is often questionable. Most studies have commonly found a non-significant increase in risk of AMI on the order of 15% to 20% for rosiglitazone compared to metformin. Our findings were similar in patients who added or switched treatment from metformin, a meaningful real- world contrast since intolerance or failure on metformin would be the most common pathway to starting a glitazone. A risk increase of 15% to 20% is clinically significant. The RECORD trial, designed to evaluate the cardiovascular safety of rosiglitazone, likely did not enroll enough patients to detect a 15% risk increase in AMI or cardiovascular death.

In our cohort of prior metformin users, adding rosiglitazone was not associated with an increased risk of AMI compared to adding a sulfonylurea, or compared to adding pioglitazone. For each of rosiglitazone and sulfonylureas separately, adding treatment with those agents was accompanied by significantly increased AMI risk after their initiation. It is unknown if the risk was increased due to worsening glycemic control which led to treatment, or if the risk was increased by more intensive treatment itself. Both hypotheses are credible and more research is needed since they have very different implications for treatment.

### Approvals

The study received ethics approval from the University of British Columbia (UBC CREB Number H02-70020). The BC Ministry of Health approved data access.
