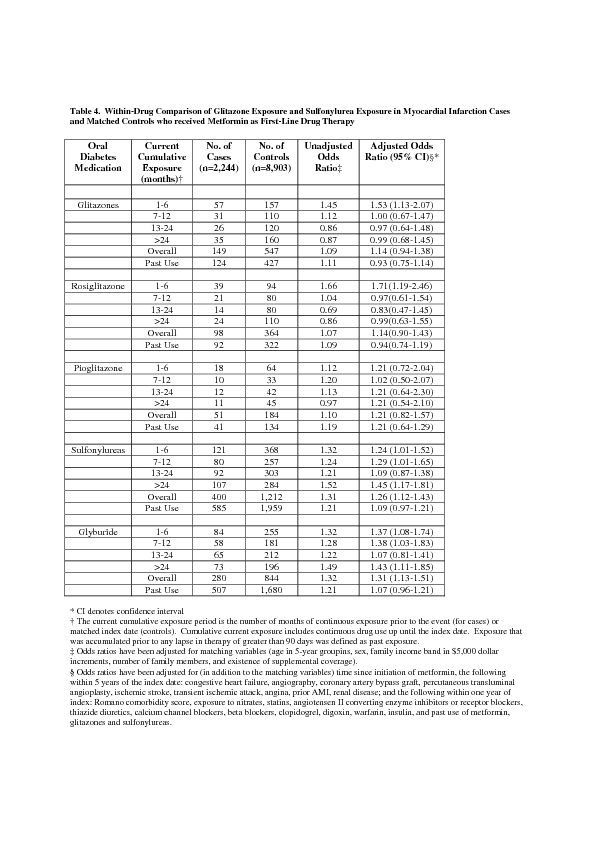# Correction: Rosiglitazone and Myocardial Infarction in Patients Previously Prescribed Metformin

**DOI:** 10.1371/annotation/3330720e-5520-4211-91f3-d3b3d20e9804

**Published:** 2010-07-29

**Authors:** Colin R. Dormuth, Malcolm Maclure, Greg Carney, Sebastian Schneeweiss, Ken Bassett, James M. Wright

The data in the columns "No. of Cases," "No. of Controls," and "Unadjusted Odds Radio" for the Rosiglitazone rows are duplicates of the equivalent data for Pioglitazone. Please view the corrected Table 4 here: 

**Figure:**